# A neck compression injury criterion incorporating lateral eccentricity

**DOI:** 10.1038/s41598-020-63974-w

**Published:** 2020-04-28

**Authors:** Tom Whyte, Angela D. Melnyk, Carolyn Van Toen, Shun Yamamoto, John Street, Thomas R. Oxland, Peter A. Cripton

**Affiliations:** 1 Orthopaedic and Injury Biomechanics Group, Departments of Mechanical Engineering and Orthopaedics, University of British Columbia, 818 W 10th Ave, Vancouver, BC V5Z 1M9 Canada; 2 International Collaboration on Repair Discoveries (ICORD), University of British Columbia, Vancouver, BC V6T 1Z4 Canada; 3 Combined Neurosurgical and Orthopaedic Spine Program, Department of Orthopaedics, University of British Columbia, Vancouver, V6T 1Z4 Canada; 4 School of Biomedical Engineering, University of British Columbia, Vancouver, BC V6T 1Z4 Canada

**Keywords:** Bone quality and biomechanics, Trauma, Experimental models of disease

## Abstract

There is currently no established injury criterion for the spine in compression with lateral load components despite this load combination commonly contributing to spinal injuries in rollover vehicle crashes, falls and sports. This study aimed to determine an injury criterion and accompanying tolerance values for cervical spine segments in axial compression applied with varying coronal plane eccentricity. Thirty-three human cadaveric functional spinal units were subjected to axial compression at three magnitudes of lateral eccentricity of the applied force. Injury was identified by high-speed video and graded by spine surgeons. Linear regression was used to define neck injury tolerance values based on a criterion incorporating coronal plane loads accounting for specimen sex, age, size and bone density. Larger coronal plane eccentricity at injury was associated with smaller resultant coronal plane force. The level of coronal plane eccentricity at failure appears to distinguish between the types of injuries sustained, with hard tissue structure injuries more common at low levels of eccentricity and soft tissue structure injuries more common at high levels of eccentricity. There was no relationship between axial force and lateral bending moment at injury which has been previously proposed as an injury criterion. These results provide the foundation for designing and evaluating strategies and devices for preventing severe spinal injuries.

## Introduction

Traumatic cervical spine injuries can be catastrophic and carry considerable societal and economic consequences, particularly when the spinal cord is involved. The estimated lifetime cost per individual traumatic spinal cord injury can be up to $3 million and the economic burden of new cases in Canada is an estimated $2.67 billion per year^1^. Road traffic crashes are the most common cause of spinal cord injury, followed by falls, violence and sports^2^. The incidence and prevalence of spinal cord injury has not decreased for many decades^3,4^ despite improvements in road safety and vehicle crashworthiness providing reductions in mortality and injuries to other body regions.

To prevent injury to the cervical spine, it is necessary to understand typical mechanisms of injury as well as the tolerance of the spine to mechanical loading. With a defined injury mechanism, defining tolerance levels involves determining how much force or acceleration the body or a part of the body can withstand before injury occurs and must consider the rate at which the force or acceleration varies with time in the biological material^5,6^. Injury criteria can then be used to design human surrogates, such as an anthropometric test device (ATD), and vehicle occupant protective systems or safety devices for falls and sports. Our current understanding of cervical spine biomechanics in axial compression has been informed by impact tests using human cadaver spines or spine segments under various boundary conditions^7–16^. These studies helped define a compressive force limit for the cervical spine of 1.75–4.8 kN^17^ but have also shown that incorporating forces and moments beyond compressive force alone (e.g. flexion/extension bending moment) is needed to fully describe the tolerance of the spine; this is because the profile of failure loads and the injury outcome in axial compression are highly dependent on the mechanism of injury and the boundary conditions of the spine at impact^8,11^.

Existing injury criteria of the cervical spine have primarily focused on load combinations of axial force and flexion-extension moments that are measured with ATDs. These notably omit consideration of failure due to coronal plane loads (i.e. lateral bending) despite the combination of compression and lateral bending being implicated as a common injury mechanism in rollover vehicle crashes^18,19^. Although a rare crash type constituting around 2.2% of crashes^20^, more than 30% of fatalities occur in rollover crashes^21^. When the most serious rollover injuries occur (maximum Abbreviated Injury Scale 3+), the spine is the second most frequently injured body region following the thorax^22^. Lateral bending may also be present to some degree in cases of compressive spinal injury documented in falls, diving into shallow water and sports including American football, rugby, ice hockey and gymnastics^23,24^. There is a need for a combined axial compression and lateral bending cervical spine injury criterion with accompanying critical tolerance values to interpret ATD data for injury risk assessment in simulated rollover crashes, falls and sports-related collisions.

A commonly-used injury criterion for the neck is the Neck Injury Criterion (Nij) which predicts injury based on the combination of axial force and sagittal plane bending moment experienced at the upper neck of the Hybrid III ATD. A lateral Nij criterion has been suggested for inertial (non-contact) neck injury risk assessment of side-facing aircraft seats and includes compressive force and lateral bending moment components^25^; however, to our knowledge, the development of this injury criterion has not been validated against biomechanical tests of combined compression and lateral bending loads.

By considering the spine as a strut or slender column, Toomey *et al*. (2013) proposed the combination of axial force and the distance of the force line of action in the sagittal plane from the central axis of the cervical spine (eccentricity) as an injury criterion, based on fundamental mechanics^14^. In a collection of inverted head and neck drop test experiments with varying sagittal plane eccentricity^14^, the eccentricity-based criterion improved injury prediction compared to axial force alone, and compared to the combination of axial force and sagittal plane bending moment (Nij). An eccentricity-based criterion has not yet been examined as a potential injury criterion for compression and coronal plane spine loading.

The lack of an established injury criterion for compression and lateral bending is due to limited biomechanical tests studying this type of load combination. Toomey *et al*. (2009) performed five inverted head and neck drop tests with small lateral eccentricities finding that the spine dynamics and tolerances were similar to purely sagittal plane dynamics^26^. The small number of tests and limited range of lateral force eccentricities prohibit determining compression and lateral bending injury criteria and corresponding tolerance values from this data set alone. Our laboratory has previously performed experiments compressing 12 three-vertebrae cervical spine segments and their intervertebral tissues at two degrees of lateral eccentricity (low and high) in a single boundary condition that allowed the superior end of the specimen to translate and rotate in the coronal plane^27,28^. These previous studies found that high eccentricity specimens experienced more soft tissue injuries, less bony fractures, less compressive force and higher lateral bending moments compared to the low eccentricity group. This was consistent with compressive spine biomechanics in sagittal plane and with the Nij injury criterion. However, tests with three additional specimens at an intermediate, medium eccentricity level in the same test conditions showed failure forces and moments inconsistent with an Nij-like criterion^29^. It was clear from this work that further experiments at varying eccentricity levels and another boundary condition were needed to define an injury criterion for the cervical spine under a laterally eccentric compression force. To address this fact, we tested a further 18 specimens under conditions that simulated a constraint on the head. Full details on these specimens are available in a separate publication^30^.

The two experimental protocols described above^27,28,30^ were compiled with the aim of determining a novel injury criterion with corresponding tolerance levels for cervical spine segments in axial compression applied with varying degrees of coronal plane eccentricity. Here, we examined the potential for a lateral Nij criterion and an eccentricity-based criterion in this load condition.

## Methods

Ethics approval for this study was provided by the University of British Columbia Human Ethics Board under certificate H04–70219 and all research was performed in accordance with relevant guidelines and regulations. Human specimens were obtained from body donation programs where informed consent was provided.

Data from a previously-published experimental program^27,28^ is included in this study. These experiments tested 12 specimens in two lateral eccentricity magnitudes of the applied compressive force (low and high) with the superior end of the specimen allowed to laterally rotate and translate (translation-free), see Table 1. For the present study, we include a further 3 specimens tested at an intermediate, medium, level of eccentricity in the same translation-free experimental conditions^29^.

**Table 1:** Specimen information and donor demographics (M = Male, F = Female, UK = unknown), assigned to three initial eccentricity groups (L = Low, M = Medium, H = High), a random bending direction (L = Left, R = Right) and two superior end conditions (lateral translation fixed and free to laterally translate). Average volumetric bone mineral density (vBMD) and average vertebral body area (VBA) are also provided. *Indicates specimens previously reported^27,28^ and ^indicates specimens in a submitted manuscript^30^.

Specimen ID	Spine Level	Eccentricity Group [Low, Medium, High]	Initial Eccentricity [mm]	Side [Left, Right]	Lateral Translation Constrained [Fixed] or Unconstrained [Free]	Sex [Male, Female]	Age [years]	Average vBMD [mg HA/cm^3^]	VBA [mm^2^]
H1318*	C5-C7	L	0.3	R	Free	F	72	620	317
H1323*	C3-C5	L	0.3	L	Free	M	UK	597	358
H1321*	C4-C6	L	0.3	R	Free	M	72	604	405
H1298*	C3-C5	L	0.3	R	Free	F	68	536	343
H1275*	C6-T1	L	0.3	L	Free	M	79	581	467
H1274*	C3-C5	L	0.2	L	Free	M	78	578	433
H1005^	C6-T1	L	1.6	L	Fixed	M	70	570	602
H1006^	C3-C5	L	1.5	R	Fixed	F	79	597	535
H1027^	C5-C7	L	1.6	R	Fixed	M	80	667	501
H1029^	C5-C7	L	1.4	L	Fixed	F	90	487	382
H1385^	C3-C5	L	1.5	R	Fixed	M	63	532	436
H1386^	C6-T1	L	1.7	L	Fixed	M	63	633	528
Low eccentricity group average (standard deviation)							74.0 (8.1)	442.3 (87.0)	583.5 (49.4)
H1045	C4-C6	M	13	R	Free	F	71	577	329
H1320	C5-C7	M	14	R	Free	M	75	665	410
H1050	C3-C5	M	14	L	Free	M	38	570	404
H1005^	C3-C5	M	15	R	Fixed	M	70	586	521
H1023^	C5-C7	M	13	L	Fixed	F	80	659	314
H1012^	C4-C6	M	15	L	Fixed	M	62	661	478
H1030^	C5-C7	M	14	R	Fixed	M	73	596	377
H1299^	C3-C5	M	15	L	Fixed	M	51	562	484
H1385^	C6-T1	M	16	R	Fixed	M	63	529	498
Medium eccentricity group average (standard deviation)							64.8 (13.2)	423.9 (75.3)	600.6 (49.4)
H1125*	C4-C6	H	40	R	Free	M	UK	523	390
H1329*	C5-C7	H	48	R	Free	M	UK	566	469
H1275*	C3-C5	H	41	L	Free	M	79	599	392
H1286*	C4-C6	H	38	L	Free	F	66	623	370
H1298*	C6-T1	H	44	R	Free	F	68	526	410
H1292*	C3-C5	H	43	L	Free	M	67	580	443
H1010^	C5-C7	H	42	L	Fixed	M	84	657	418
H1014^	C3-C5	H	39	R	Fixed	M	48	607	374
H1014^	C6-T1	H	49	R	Fixed	M	48	582	445
H1019^	C5-C7	H	44	R	Fixed	M	85	611	421
H1028^	C4-C6	H	43	L	Fixed	M	75	553	438
H1386^	C3-C5	H	38	L	Fixed	M	63	608	401
High eccentricity group average (standard deviation)							68.3 (13.1)	414.3 (30.5)	586.3 (39.6)

Data from another experimental series^30^ is also included in this study. In these experiments, 18 specimens were tested at three lateral eccentricity magnitudes of the applied compressive force (low, medium and high) in an end constraint allowing the superior-most vertebra to laterally rotate but not translate (translation-fixed).

The following sections describe the specimens from each experimental series, the dynamic test protocol, injury identification and severity assessment, and analysis carried out for this study.

### Translation-fixed experiment specimens

Eighteen human cadaveric subaxial cervical functional spinal units (FSUs), free from tumours, fusion or evidence of previous spinal surgery, were prepared by dissection. Prepared FSUs consisted of three vertebrae with bones, ligaments and intervertebral discs preserved. The inferior and superior vertebrae were potted in polymethylmethacrylate in a posture aligning the middle vertebral body parallel to the ground using a custom potting rig and levelling lasers. This posture was intended to align the compression force vector parallel to the long axis of the spine. The compressive force was applied at 1% or 5% (low), 50% (medium) and 150% (high) of the specimen vertebral body width from the midline in the coronal plane. Specimens were randomly assigned either left or right lateral bending. Volumetric bone mineral density (vBMD) was obtained by high-resolution CT scanning the specimens (Xtreme CT, Scanco Medical, Scanco software µCT Evaluation Program V6.0, Brüttisellen, Switzerland, resolution 246 µm). Vertebral body area (VBA) was calculated by measuring the sagittal and coronal plane diameters and assuming the vertebral body to be an ellipse. Cohorts in each eccentricity group were selected attempting to provide a similar average age, VBA and vBMD per group. All specimen details and test conditions are summarised in Table 1. The average (standard deviation) age and vBMD for the cohort was 69.3 (11.8) years and 589.2 (44.9) HA/cm^3^, respectively.

### Dynamic “impact” testing

Dynamic testing was carried out on each specimen to simulate loading that would occur to a spine segment during a head-first impact event. One loading run was carried out on each specimen following flexibility testing, described in detail elsewhere^27,30^, which pre-conditioned the specimen. The prepared specimen was inserted into a custom rig incorporated into a materials testing system (Model 8874, Instron, Norwood, Massachusetts). The actuator was attached to the rig, rather than impacting the rig, and applied axial compression at a nominal rate of 500 mm/s aligned at the predetermined lateral eccentricity (distance in the coronal plane from the centre of the spine) of each specimen. While head-first impacts that result in cervical spine injuries occur at an impact speed of 3 m/s^12^, for segment testing it is appropriate to scale down this velocity from what is seen across the whole cervical spine^31,32^. To account for physiologic, non-injurious lateral bending rotation that can occur at higher magnitudes of lateral force eccentricity, the actuator was displaced further with increasing eccentricity, being 20%, 22% and 40% of the specimen height for the low, medium and high eccentricity groups respectively. The specimen height was the vertical distance from the superior-most aspect of the superior vertebral body to the inferior-most aspect of the inferior vertebral body in the potting orientation (i.e. the total height of the vertebral bodies of the three vertebra segment). An initial 20% displacement was selected as an appropriate level of compression for spine segments in pure axial compression based on previously published segment compression testing shown to result in injury^8,33,34^. To estimate the additional compression needed to account for physiologic bending, we estimated the expected lateral rotations of spine segments at each proposed eccentricity level. To do this, we calculated the expected lateral bending that would occur under 150 N of eccentrically applied force, corresponding to the approximate segmental compression force in a relaxed posture^35^. Previously reported segment single-side range of motion in lateral bending^36,37^ were used to estimate rotational segment stiffness of 9.6 Nm/rad. With typical vertebral segment measurements, we estimated physiologic rotations of 1°, 12° and 37° under this eccentric 150 N force, corresponding to additional vertical actuator displacements of 0%, 2% and 20% (totalling 20%, 22% and 40% displacement) for the low, medium and high eccentricity groups respectively. Pilot testing confirmed the appropriateness of these target displacements for producing injury at each eccentricity level. At the end of the compression ramp, the actuator paused for 0.1 s and then returned to the starting position at a rate of 50 mm/s. The dynamic test event was captured by high speed cameras (v9 at 6400 Hz, v12.1 s at 18000 Hz, Vision Research, Wayne, NI, USA). A six-axis load cell beneath the specimen (4366 K, Denton ATD, Inc., Rochester Hills NJ) sampled forces and moments at 50 kHz.

**Figure 1:**
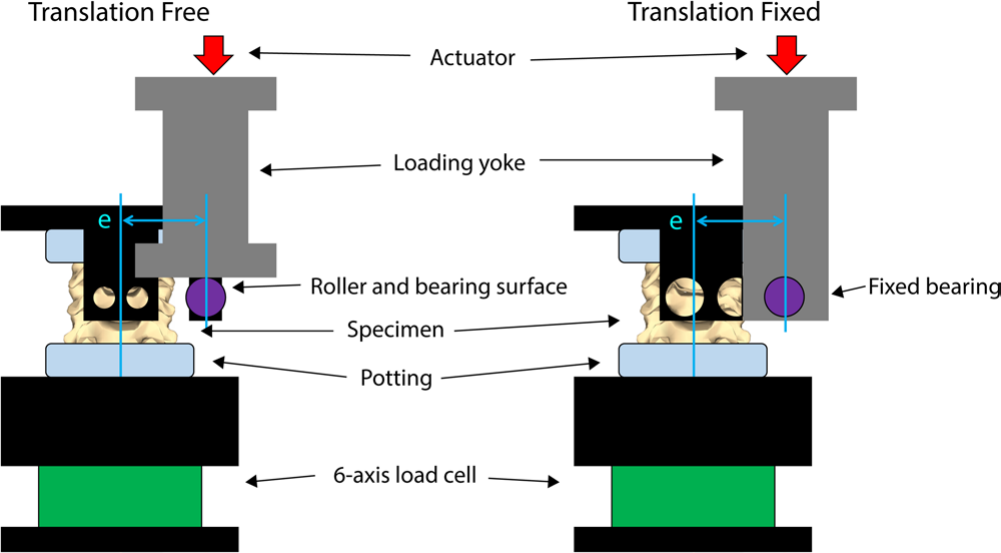
Dynamic “impact” test setup looking toward the anterior side of the specimen for the end constraint allowing lateral translation and rotation of the superior end of the specimen (left, Translation Free) and the end constraint allowing only lateral rotation and not translation (right, Translation Fixed) showing the load applied at a predefined initial eccentricity (e).

The difference in experimental setup between the translation-fixed and the translation-free protocols is shown in Fig. 1. In the translation-fixed experiments, a fixed bearing housed within the custom rig constrained lateral translation but allowed the superior end of the specimen to rotate laterally. In the translation-free experiments, the actuator contacted a roller at a bearing surface, allowing the roller (and superior end of the specimen) to rotate and translate along the bearing surface.

### Injury identification and severity assessment

The time of injury was determined from the high-speed video footage. Injury was defined and identified by a sudden unexpected change in appearance on the surface of the spine occurring within 10 video frames (~0.6 ms) indicating initiation of soft tissue failure or large, more than expected motion of one structure relative to an adjacent structure between video frames indicating initiation of bone fracture. In some cases of soft tissue injury, injury was identified in the video footage and tracked back to the first evidence of injury initiation to define the time of injury. Example high speed video clips of soft and hard tissue injuries are provided in supplementary material. Time of injury was recorded independently by two authors and disagreements were resolved by discussion, reaching consensus in all cases. The earliest point of identified tissue failure, so-defined, was used as the time of injury and represents the initiation of injury in each specimen.

A spine surgeon (JS, SY) dissected the specimens and diagnosed and graded the injuries as described in detail previously^27^. Briefly, 134 structures on and between each of the three vertebrae were examined and a score (0–2) was assigned to each structure based on whether it was intact (0), partially injured (1) or completely injured (2). For this study, the Abbreviated Injury Scale (AIS) 2005 (update 2008) was used to assign a severity score to each injury of each specimen. The maximum AIS (MAIS) score for each specimen (most severe injury) was recorded. Since the degree of anterior height loss of each specimen was not recorded, all vertebral body fractures were graded as AIS 2 (vertebral body not further specified). Partially injured intervertebral discs were graded as AIS 2 (disc herniation) while completely injured intervertebral discs were graded as AIS 3 (disc rupture).

### Data analysis

All data were processed in MATLAB (R2016a, MathWorks Inc., Natick, MA, USA).

Loads were calculated at time of injury. Forces and moments were low-pass filtered with a 4^th^ order Butterworth profile (900 Hz cut-off frequency for forces and 150 Hz cut-off frequency for moments) and 60 Hz notch filtered. Loads were transformed from the load cell origin to the centre of the inferior-most intervertebral disc using sagittal and coronal plane x-rays of the potted specimen taken prior to testing. Analysis was not carried out at the superior-most intervertebral disc.

**Figure 2:**
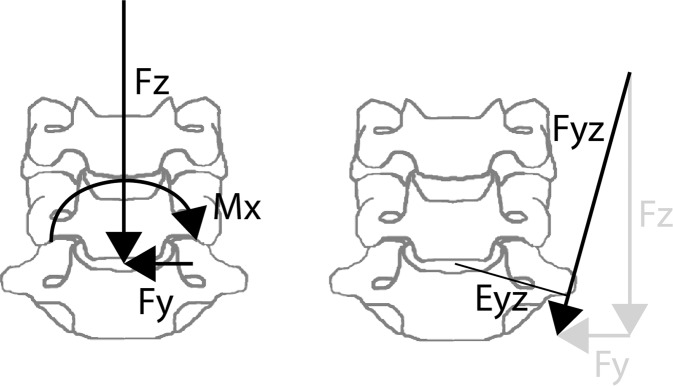
Compression force (Fz), contralateral lateral shear force (Fy) and ipsilateral lateral bending moment (Mx) coronal plane loads on the inferior disc for a specimen subjected to a left eccentric compression force (left) and calculated coronal plane resultant force, Fyz, and eccentricity, Eyz (right).

Coronal plane eccentricity (Eyz) at injury was calculated from the lateral bending moment (Mx) and resultant force (Fyz) in the coronal plane at injury according to Eq. (1). These loads are pictured on a FSU in Fig. 2.

1 $$Eyz=\frac{Mx}{\sqrt{F{y}^{2}+F{z}^{2}}}$$

### Revisited translation-free experiments

The previously reported translation-free experiments^27,28^ were revisited for this study as follows:

Time of injury initiation was revised from previously using acoustic emission sensors to using high-speed video injury identification for consistency with the translation-fixed specimens. The difference in time of injury determined by acoustic emission sensors compared to video evidence of injury was found to be less than 0.5 ms for low eccentricity specimens and approximately 3 ms for high eccentricity specimens, on average^28^. In one case (H1286) high-speed video was not captured and so time of injury was based on acoustic emission sensors using the previously reported method^28^.

Further, the translation-free specimens were assigned an AIS severity score based on the injury outcome and coronal plane eccentricity was calculated from the measured loads.

### Injury criterion assessment

Two relationships were investigated as potential neck compression injury criteria incorporating coronal plane forces:

- Axial compression force and lateral bending moment at injury (a Nij-like criterion).
- Resultant coronal plane force and calculated coronal plane eccentricity, see Eq. (1), at injury (an eccentricity criterion).

Multiple linear regression was performed for these two combinations of variables in GraphPad Prism version 8 for Windows (GraphPad Software, La Jolla, California, USA) incorporating specimen age, VBA and vBMD since these variables influence failure loads^28,38^. A p-value of 0.05 was considered significant. Failure loads differ by sex due to factors beyond age, size and bone density^39^, hence male and female specimens should be analysed as separate populations for biomechanical tolerance^11^. Therefore, linear regressions were also performed for male and female specimens separately.

## Results

**Figure 3:**
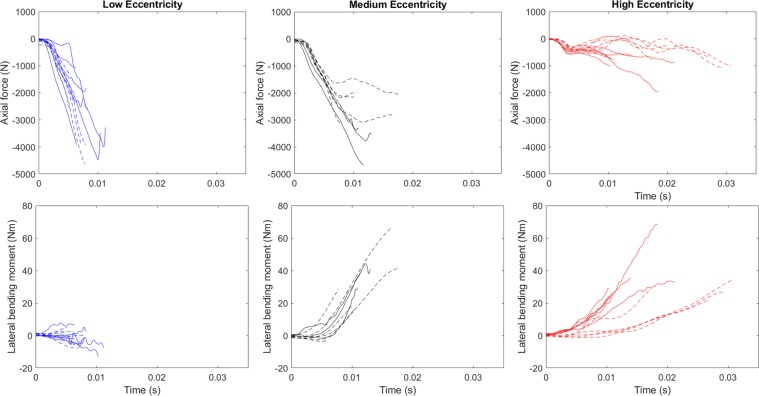
Axial force-time response (top) and lateral bending moment-time response (bottom) for the low (left), medium (centre) and high (right) initial eccentricity specimen groups. Solid lines indicate the unconstrained lateral translation end condition and dashed lines indicate the constrained lateral translation end condition. Positive force is tension and positive lateral bending moment is to the ipsilateral side.

Predominant forces and moments experienced by each specimen, and calculated coronal plane eccentricity at time of injury initiation are summarised in Table 2. Compressive axial force was the dominant force for all specimens, as expected, with an average magnitude 99.6% of resultant coronal plane force (range 94.8–100.0%). Lateral shear force magnitude was 6.2% of resultant coronal plane force, on average, (range 0.1–31.9.%). Lateral bending moment was the largest contributor to resultant moment for all of the medium and high initial lateral eccentricity specimens, but only one third of the low initial eccentricity specimens (4/12) with flexion-extension moments (7/12) or axial rotation moments (1/12) dominating for the other specimens. Lateral bending moment magnitude was on average 77.0% of resultant moment (range 0.2–98.6%). Flexion-extension moment was on average 39.4% of resultant moment (range 2.8–99.8%) and axial rotation moment on average 28.8% of resultant moment (range 1.9–82.8%). Axial force-time and lateral bending moment-time curves for all specimens are shown in Fig. 3.

**Table 2:** Specimen forces, moments and calculated coronal plane eccentricity at time of injury initiation. *Indicates specimens previously reported, with values now corresponding to a revised time of injury except for specimen H1286^27,28^. ^Indicates specimens in a submitted manuscript^30^. **No high speed video was collected for specimen H1286 so values remain relative to indications of injury as determined by acoustic emission sensors reported previously^28^.

Specimen ID	Spine Level	Initial Eccentricity [mm]	Lateral Translation Constrained [Fixed] or Unconstrained [Free]	Ipsilateral Lateral Shear Force Fy [N]	Force Fz [N] (Tension +ve)	Ipsilateral Lateral Bending Moment Mx [Nm]	Calculated Coronal Plane Eccentricity Eyz [mm]	Time of Injury [ms]
H1318*	C5-C7	0.3	Free	−21.0	−1064.5	−2.0	−1.9	6.3
H1323*	C3-C5	0.3	Free	−101.5	−3281.3	−7.2	−2.2	11.3
H1321*	C4-C6	0.3	Free	−43.6	−2580.4	2.8	1.1	8.3
H1298*	C3-C5	0.3	Free	−72.4	−1829.6	−1.1	−0.6	8.0
H1275*	C6-T1	0.3	Free	−239.5	−3493.2	−12.4	−3.5	10.4
H1274*	C3-C5	0.2	Free	−87.4	−3915.4	7.0	1.8	6.4
H1005^	C6-T1	1.6	Fixed	−110.0	−2555.8	5.0	2.0	5.2
H1006^	C3-C5	1.5	Fixed	−4.5	−4630.1	0.1	0.0	7.8
H1027^	C5-C7	1.6	Fixed	−97.2	−3882.4	−2.9	−0.7	8.0
H1029^	C5-C7	1.4	Fixed	−10.0	−1294.2	2.9	2.2	5.2
H1385^	C3-C5	1.5	Fixed	−127.7	−3923.6	−7.5	−1.9	7.2
H1386^	C6-T1	1.7	Fixed	−57.8	−2300.4	3.0	1.3	6.1
H1045	C4-C6	13	Free	−182.0	−3332.5	29.6	8.9	10.9
H1320	C5-C7	14	Free	−93.7	−3546.3	40.8	11.5	13.0
H1050	C3-C5	14	Free	−159.1	−4676.2	40.7	8.7	11.7
H1005^	C3-C5	15	Fixed	−92.1	−1821.1	−2.9	−1.6	5.8
H1023^	C5-C7	13	Fixed	−247.8	−3156.1	28.7	9.1	7.8
H1012^	C4-C6	15	Fixed	−517.9	−2168.3	19.1	8.6	10.1
H1030^	C5-C7	14	Fixed	−159.6	−1996.4	16.7	8.3	10.5
H1299^	C3-C5	15	Fixed	−206.6	−2828.3	66.6	23.5	16.5
H1385^	C6-T1	16	Fixed	−68.6	−2049.0	41.5	20.3	17.8
H1125*	C4-C6	40	Free	−45.9	−708.1	22.9	32.3	10.5
H1329*	C5-C7	48	Free	33.4	−621.6	35.6	57.3	13.9
H1275*	C3-C5	41	Free	−47.0	−1967.9	68.7	34.9	18.4
H1286*	C4-C6	38	Free	−32.6**	−1019.8**	29.4**	28.8**	10.3**
H1298*	C6-T1	44	Free	−44.8	−892.8	32.7	36.6	21.2
H1292*	C3-C5	43	Free	−35.4	−787.2	25.5	32.3	11.1
H1010^	C5-C7	42	Fixed	−38.4	−260.8	12.2	46.2	19.9
H1014^	C3-C5	39	Fixed	−126.3	−988.8	34.5	34.6	30.8
H1014^	C6-T1	49	Fixed	−78.2	−668.9	21.9	32.5	26.9
H1019^	C5-C7	44	Fixed	−11.0	−772.0	29.0	37.5	17.4
H1028^	C4-C6	43	Fixed	−113.9	−338.2	12.8	35.7	9.1
H1386^	C3-C5	38	Fixed	−72.5	−1070.8	26.8	25.0	29.1

Major injuries to each specimen following the entire loading event are listed in Table 3. The low eccentricity specimen group sustained the highest proportion of all hard tissue structure injuries compared to the medium and high eccentricity groups whereas the high eccentricity group sustained the highest proportion of all soft tissue structure injuries compared to the medium and low eccentricity groups, see Table 4. Given the majority of complete intervertebral disc injuries were sustained by specimens subjected to high eccentricity compression, this group had a higher proportion of AIS 3 injuries, followed by medium eccentricity specimens and then low eccentricity specimens who predominantly sustained injuries classified as AIS 2 (Table 3).

**Table 3:** Resulting specimen maximum abbreviated injury scale score and completely injured structures from the entire dynamic loading event. *Indicates specimens previously reported^27,28^. ^Indicates specimens in a submitted manuscript^30^.

Specimen ID	Spine Level	Initial Eccentricity [mm]	Lateral Translation Constrained [Fixed] or Unconstrained [Free]	Maximum Abbreviated Injury Scale Score	Completely Injured Structures (V1 superior, V2 middle, V3 inferior): vertebral body (VB), end plate (EP), facet joint (FJ), pedicle (PED), transverse process (TP), lamina (LAM), lateral mass (LM), uncinate process (UP), spinous process (SP), facet capsule (FC), anterior longitudinal ligament (ALL), posterior longitudinal ligament (PLL), intervertebral disc (IVD), ligamentum flavum (LF), interspinous ligament (ISL), supraspinous ligament (SSL).
H1318*	C5-C7	0.3	Free	2	V1: EP (inf), VB; V2: LM, TP; V3: EP (sup), UP, PED
H1323*	C3-C5	0.3	Free	2	V1: VB; V2: EP (sup), VB, LAM, SP
H1321*	C4-C6	0.3	Free	3	V1: TP; V1/2: IVD; V2: EP (sup), VB
H1298*	C3-C5	0.3	Free	2	V2: EP (sup), LAM, SP, UP
H1275*	C6-T1	0.3	Free	2	V2: EP (sup & inf), VB; V3: FJ (sup)
H1274*	C3-C5	0.2	Free	3	V1: VB; V1/2: IVD; V2:FJ (sup), TP; V3: VB
H1005^	C6-T1	1.6	Fixed	2	V2: VB, FJ (inf); V2/3: FC; V3: EP (sup), VB, PED, UP
H1006^	C3-C5	1.5	Fixed	2	V1: EP (inf), VB, LM, FJ (inf), PED, TP; V1/2: FC; V2: LAM, LM
H1027^	C5-C7	1.6	Fixed	2	V1/2: FC; V3: EP (sup), VB, LAM, LM
H1029^	C5-C7	1.4	Fixed	2	V1: PED: V2: EP (sup & inf), VB, LAM; V3: TP, PED, LAM, LM, UP
H1385^	C3-C5	1.5	Fixed	3	V1: EP (inf); V1/2: ALL, IVD, LF; V2: EP (sup), VB, LAM, TP; V2/3: FC; V3: FJ (sup)
H1386^	C6-T1	1.7	Fixed	2	V2: EP (sup), VB, TP; V3: EP (sup), VB, FJ
H1045	C4-C6	13	Free	2	V3: EP (sup), VB
H1320	C5-C7	14	Free	2	V1: EP (inf), VB; V2: EP (sup), VB, PED; V3: LM
H1050	C3-C5	14	Free	2	V2: EP (inf); V3: EP (sup)
H1005^	C3-C5	15	Fixed	3	V2/3: ALL, PLL, IVD, FC
H1023^	C5-C7	13	Fixed	3	V1: TP, PED; V1/2: IVD, FC; V3: LAM
H1012^	C4-C6	15	Fixed	3	V2/3: IVD, FC, LF
H1030^	C5-C7	14	Fixed	3	V1: LM; V2: EP (sup); V2/3: IVD, FC; V3: EP (sup), VB, LAM, LM
H1299^	C3-C5	15	Fixed	3	V1: EP (inf); V1/2: IVD, FC; V2: EP (sup & inf), VB; V2/3: FC, LF
H1385^	C6-T1	16	Fixed	3	V2: EP (inf); V2/3: IVD, FC
H1125*	C4-C6	40	Free	3	V2: TP; V2/3: FC, IVD
H1329*	C5-C7	48	Free	3	V1: EP (inf); V2/3: FC, LF, ISL, SSL, IVD; V3: FJ, LM, TP
H1275*	C3-C5	41	Free	3	V2: EP (inf) VB, TP; V2/3: FC, LF, ISL, SSL, ALL, PLL, IVD
H1286*	C4-C6	38	Free	3	V1/2: FC, LF, ISL, SSL, ALL, IVD; V2: EP (sup), PED, LAM, TP, LM
H1298*	C6-T1	44	Free	2	V1: FJ (inf); V2: FJ (sup), EP (inf); V2/3: FC, LF; V3: EP (sup), TP
H1292*	C3-C5	43	Free	3	V2: EP (inf); V2/3: FC, LF, ALL, PLL, IVD; V3: EP (sup)
H1010^	C5-C7	42	Fixed	3	V1: EP (inf), VB, TP, PED, FJ; V1/2: ALL, IVD, FC; V2: TP
H1014^	C3-C5	39	Fixed	3	V1/2: IVD, FC
H1014^	C6-T1	49	Fixed	3	V2/3: IVD, FC
H1019^	C5-C7	44	Fixed	3	V2: EP (inf), VB; V2/3: ALL, PLL, IVD, FC, LF
H1028^	C4-C6	43	Fixed	2	V1: EP (inf), VB; V1/2: ALL, FC, LF
H1386^	C3-C5	38	Fixed	3	V1/2: FC; V2/3: ALL, PLL, IVD, FC, LF; V3: FJ (sup)

**Table 4:** Proportion of specimens in each failure group with complete injury to each type of hard and soft tissue structure.

Eccentricity Group	Proportion of hard tissue structures injured (%)									Proportion of soft tissue structures injured (%)						
	End plate	Vertebral body	Uncinate process	Transverse process	Lateral mass	Facet joint	Pedicle	Lamina	Spinous process	Intervertebral disc	Facet capsular ligaments	Anterior longitudinal ligament	Posterior longitudinal ligament	Ligamentum flavum	Interspinous ligament	Supraspinous ligament
**Low**	92	92	33	58	33	42	33	50	17	33	8	0	8	25	0	0
**Medium**	67	44	0	11	22	0	22	22	0	56	11	11	22	67	0	0
**High**	67	33	0	50	17	33	17	8	0	100	58	33	67	83	25	25

**Figure 4:**
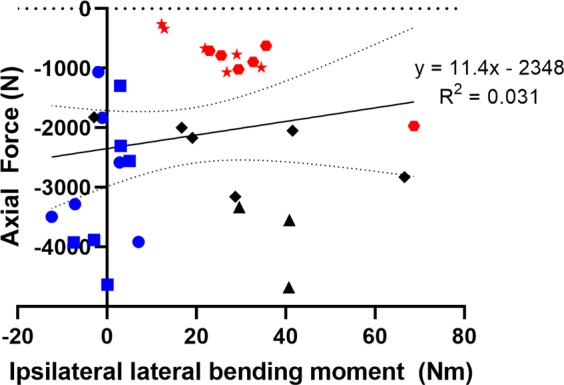
Lateral bending moment (ipsilateral is positive) and axial force (tension is positive) at failure for each specimen in the following initial eccentricity and end constraint groups: low-fixed (blue square), low-free (blue circle), medium-fixed (black rhombus), medium-free (black triangle), high-fixed (red star), high-free (red hexagon). Previously published data^27,28^, with a revised time of injury, are the low-fixed and high-fixed specimens. The linear regression line (solid line), equation, 95% confidence interval (dotted lines) and coefficient of determination are shown for the combined data.

The multiple regression equation relating compressive axial force to lateral bending moment, age, VBA and vBMD was not statistically significant (p = 0.774, R^2^ = 0.067). Axial compression force was not related to lateral bending moment for all specimens (p = 0.326, R^2^ = 0.031), see Fig. 4, nor for male (p = 0.328, R^2^ = 0.042) and female (p = 0.876, R^2^ = 0.004) specimens separately.

**Figure 5:**
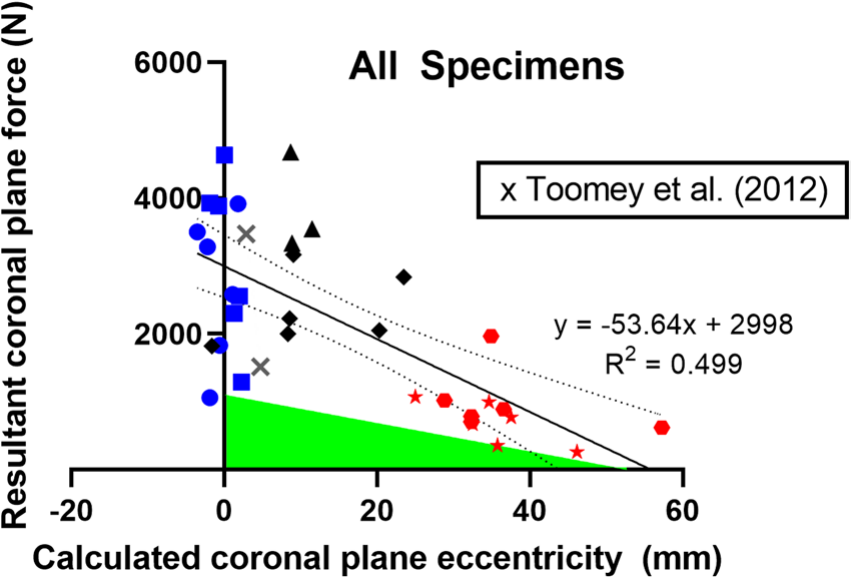
Coronal plane eccentricity at injury calculated from the lateral bending moment and coronal plane resultant force at injury plotted with the magnitude of coronal plane resultant force at injury for each specimen in the following initial eccentricity-end constraint groups: low-fixed (blue square), low-free (blue circle), medium-fixed (black rhombus), medium-free (black triangle), high-fixed (red star), high-free (red hexagon). The linear regression line (solid line), equation, 95% confidence interval (dotted line) and coefficient of determination are shown for the combined data of this study. Black crosses show data from compression tests with lateral eccentricity described by Toomey *et al*. (2012). The shaded green area indicates a region of coronal plane eccentricity and force in which no specimens initiated injury in the current data.

Larger coronal plane force was statistically significantly predicted by the multiple regression model, F(4,25) = 6.734, p = 0.0008, R^2^ = 0.519 incorporating coronal eccentricity at failure, age, VBA and vBMD. Only coronal plane eccentricity at failure added statistically significantly to the model (p < 0.0001), while age (p = 0.331), VBA (p = 0.538) and vBMD (p = 0.370) were not statistically significant. Removing the non-significant independent variables, larger resultant coronal plane force remained associated with smaller calculated coronal plane eccentricity at injury (p < 0.001, R^2^ = 0.499) for all specimens, see Fig. 5, and for male specimens (p < 0.001, R^2^ = 0.636) while not for female specimens (p = 0.303, R^2^ = 0.175), likely due to small specimen numbers.

Considering a linear relationship between coronal plane force and eccentricity, a region in Fig. 5 was shaded green to indicate a cut-off where the specimens with the lowest tolerance first initiated injury. Hence, there was no injury initiation in the region with intercepts of 1103.8 N of coronal plane resultant force and 52.8 mm of coronal plane eccentricity.

**Figure 6:**
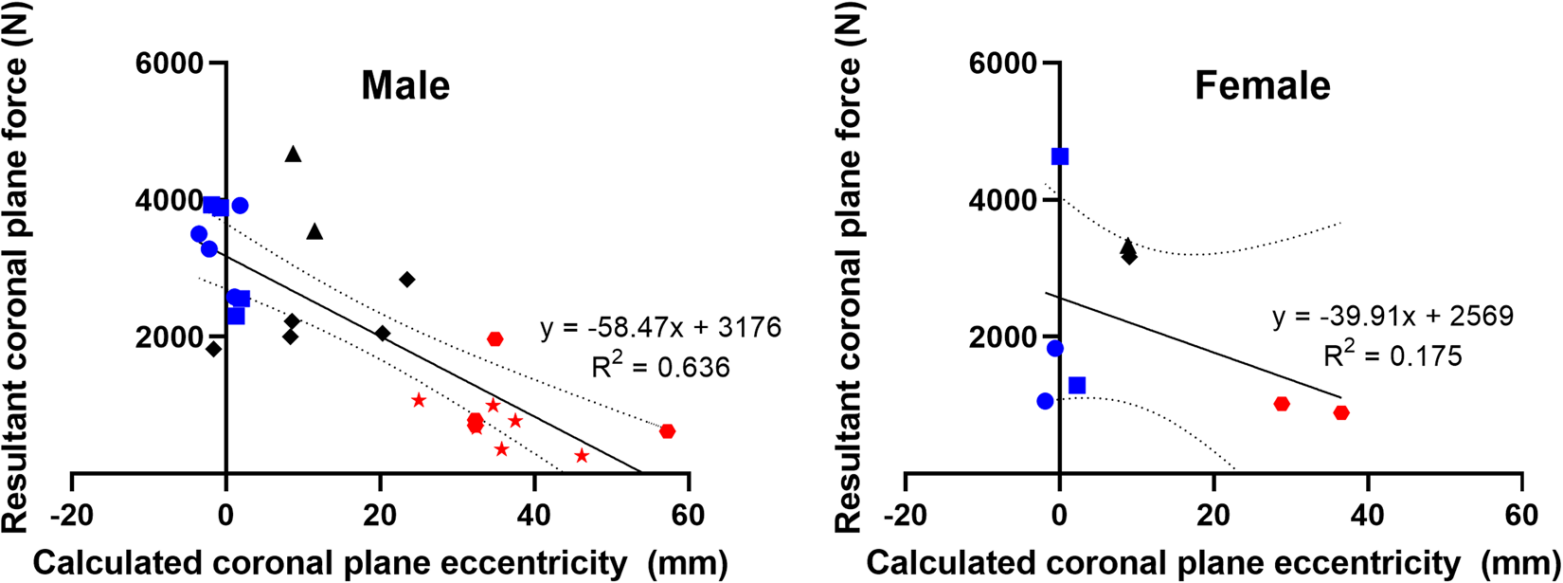
Coronal plane eccentricity and resultant coronal plane force for male specimens (left) and female specimens (right). Initial eccentricity-end constraint groups are indicated as follows: low-fixed (blue square), low-free (blue circle), medium-fixed (black rhombus), medium-free (black triangle), high-fixed (red star), high-free (red hexagon). Linear regression lines (solid lines), equations, 95% confidence intervals (dotted lines) and coefficients of determination are shown.

The intercepts for the linear regression relating coronal plane force and eccentricity for male specimens are 54.3 mm of coronal plane eccentricity and 3176 N of resultant coronal plane force at injury, see Fig. 6. For female specimens the corresponding intercepts are 64.4 mm and 2569 N, see Fig. 6.

## Discussion

This study is the first to conduct detailed evaluation and synthesis of compressive cervical spine injury as a combination of coronal plane loads based on biomechanical tests of human specimens. A linear relationship was identified between resultant coronal plane force and eccentricity at failure. These findings have significant implications, providing the foundation for designing an ATD capable of predicting cervical spine injury in vehicle rollovers, falls and sports and the development of injury prevention strategies in these scenarios.

Importantly, the results of this study suggest that a coronal plane Nij neck injury criterion relating compressive force and lateral bending moment is not appropriate for predicting injury of cervical spine segments and could lead to erroneous conclusions for neck injury risk. A previously proposed lateral Nij^25^ was based on Nij formulated for combined axial force and flexion-extension moments and assumes a decreasing compressive force tolerance with increasing lateral bending moment, a relationship that was not present for the cadaveric segment tests performed for this study. There appears to be a high-eccentricity specific linear relationship of increasing axial force and bending moment at injury (red points in Fig. 4), which is related to the initial applied force eccentricity as also shown by Van Toen *et al*.^28^. Without knowing the initial eccentricity of the applied force such as in the case of assessing injury risk using a mechanical surrogate, this relationship has limited utility as an injury criterion.

The relationship of spine failure as a combination of coronal plane force and eccentricity appears to distinguish between different patterns of spine injury in compression and lateral bending. Specimens subjected to compression with low initial lateral eccentricity failed with a calculated coronal plane eccentricity between −3.5 and 2.2 mm (Table 2, Fig. 5) and resulted in an injury profile from the entire loading event typically involving more hard tissue structures (Tables 3 and 4) indicative of pure axial compression of the spine^9,13,40^. Given the loads developed and resulting injuries, the compressive force appears to be the dominant contributor to injury in this loading mode. High initial eccentricity specimens produced coronal plane eccentricity values at failure of between 25.0 and 57.3 mm and exhibited fewer hard tissue injuries and more soft tissue injuries (Tables 3 and 4) compared to low eccentricity specimens. This suggests the lateral bending moment component is playing a larger role in failure of the spine in this mode of injury in the same way flexion-compression produces more soft tissue injuries than pure axial compression^13,40^. With the exception of one specimen with a coronal plane eccentricity at failure of −1.6 mm, all medium initial eccentricity specimens exhibited coronal plane failure eccentricity values between the low and high eccentricity specimen groups (8.3–23.5 mm). The injury profile of the medium eccentricity specimens varied between resembling specimens in the low and high eccentricity groups (Table 3) with proportions of hard and soft tissue structure injuries typically between the high and low eccentricity groups (Table 4). With more medium initial eccentricity data, it may be possible to define robust criteria for determining whether a measured load profile is more likely to result in spinal injury representative of a compression type failure or lateral bending type failure.

Although the eccentricity-based relationship identified here was not significant for female sex, likely due to low specimen numbers, the linear regressions indicate a reduced compressive force tolerance for female specimens compared to male specimens as expected from previous cervical spine tolerance studies. The forces generated during compression of spinal segments in this study are most appropriately compared to compression of the aligned spine, such as the conditions applied by Pintar *et al*. (1995)^13^. Consequently, the linear regression intercept of resultant coronal plane force, which was overwhelmingly dominated by axial compression, for male specimens of 3176 N compares favourably (within one standard deviation) to the mean compressive failure force of 3810 ± 970 N for 11 previously reported male specimens^13^, as does the same intercept for female specimens of 2569 N in this study to the mean compressive failure force of 2300 ± 1100 N for 8 previously reported female specimens^13^.

There is very scarce experimental data concerning axial cervical spine compression with lateral bending beyond that conducted in our laboratory and included in this study^27,28,30^. One other test series dropped five cadaveric head and neck specimens in an inverted posture onto a laterally angled surface or onto a flat surface in a laterally bent posture^26^. Data applicable for comparison to the results of the present study are available for two tests^41^ and these are plotted in Fig. 5. The location of these points indicates they are comparable to the low or medium eccentricity specimens of the current study. For these full spine impact experiments, Toomey documented that one specimen sustained lateral mass, facet, pedicle and lamina fractures while the other sustained facet, pedicle and lamina fractures. These injuries are most prevalent among the low initial eccentricity specimen group in this study (Table 4) although the most common injuries in the low eccentricity group (end plate and vertebral body fractures) did not occur. Note that the sagittal plane posture of the spine in these tests, relative to the compressive force vector, differed to that applied to the specimens in the current study. In the Toomey study, the complete cadaveric neck specimens were oriented in a neutral posture. More full spine drop tests and at larger lateral force eccentricities are needed to further evaluate the coronal plane force and eccentricity criterion for head-first impacts of the full cervical spine.

Further work is also needed to establish whether the eccentricity-based injury criterion is appropriate for use with any current ATD, also called a crash test dummy. A typical ATD is unable to replicate the failure response of cadaveric specimens so peak ATD load responses are often used for injury risk evaluation rather than loads at injury which are used for cadavers. Use of the coronal plane force and eccentricity failure relationship could examine the neck load response over time and predict injury if the linear regression line is crossed. Calculating eccentricity over time can result in large values when coronal plane resultant force is small so a minimum force, such as 250 N, could be recommended noting that the lowest coronal plane force at injury initiation in this dataset was 263.7 N. An ATD neck would also need to respond like human specimens in combined compression and lateral bending for direct application of the injury criterion and accompanying critical tolerance values described here. Published spine compression-flexion impact tests performed with the Hybrid III ATD to match cadaveric experiments found that peak eccentricities measured with the Hybrid III had a much smaller range than eccentricity values at injury for cadaveric specimens indicating a stiffer response of the ATD neck and limiting development of an eccentricity criterion for axial compression and sagittal plane bending moments^41^. The results of this study could inform development of a new physical surrogate for axial compression that can accommodate a larger eccentricity range for sagittal and coronal plane loads.

As with any investigation of this nature, there are a number of limitations to this study. First is the use of FSUs rather than the whole spine. Compression of the whole spine has been established to result in complex kinematics and often involves buckling that induces different mechanical loads at various segments along the spine^10–12^. We chose to use spine segments to reduce this complexity and understand and define the tolerance of segments to coronal plane loads. In doing so, any complex buckling response of the spine that may have occurred due to lateral load eccentricity could not be studied. Using spinal segments also allowed us to locate all structures that rotated with the specimen as close as possible to the load cell which was essential to minimise inertial effects induced by the experimental setup^42^. Inertial loads may be introduced by the boundary conditions experienced in impact testing complete cadaver spines or an ATD in axial loading and so the measured kinetics in these conditions should be carefully analysed in order to compare results to the loads reported in this study.

The end constraints applied to the specimens in this study were a simplification of real world axial loading. While we applied two different superior end conditions to different specimen groups, there were constraints to the superior end in both our setup conditions that may not be present in typical injurious compression of the full spine, including anterior translation, anterior rotation and axial rotation constraints, and these may have contributed to forces and moments measured at injury. The different applied end constraints appeared to have little effect on the major loads developed in the low eccentricity group while in some cases the axial force and lateral bending moment showed differences by end condition in the medium and high eccentricity groups. For instance, the slope of the axial force-time curve immediately before time of injury was typically steeper for the unconstrained lateral translation group at medium and high eccentricity. Furthermore, unconstrained lateral translation specimens generally developed lateral bending moments sooner and had shorter time to injury than laterally constrained specimens. In future testing of complete spines, any effects of the superior end constraints compared to real-world head-first impacts could be determined, along with whether the tolerance relationship observed in this study exists with fewer constraints.

All spine segments were tested in a posture aligning the spine parallel to the eccentric compressive force vector however head-first impacts in real scenarios such as rollover vehicle crashes are likely to produce complex loading situations to the spine with variable compressive force orientation^43,44^. The spine is a multi-articulated column with many possible postures and human individuals themselves adopt highly variable postures when upright and inverted^45,46^. The chosen posture of the spine and eccentrically applied loads for this study were selected to ensure consistency in the test setup and to allow comparison of the effect of load eccentricity. The test setup was devised to help facilitate comparison of the tested human specimens to finite element human body model segments of the cervical spine. Also, positional differences from this posture are likely to be smaller at a segment level than in looking at whole cervical spine angles in testing complete cervical spines.

Identification of injury in this study utilised high-speed video footage. While previous work has shown small differences in injury initiation time determined by acoustic emission sensors, local force peaks, local moment peaks and high-speed video in this test setup^28^, this visual method of determining injury initiation provides the possibility that some injury is missed (for example rupture of the ligamentum flavum) which may be indicated by local force or moment peaks. There is also a level of subjectivity in defining the time of injury visually which was minimised by using two independent observers.

In this analysis, links are drawn between forces and moments at the time of injury initiation and the resulting injuries from the entire loading event. This assumes that loading of the spine continues after injury initiation whereas if the loading event were to stop at the time of injury initiation, the injury pattern would be likely be very different in most cases. In an injurious head-first impact event, loading continues beyond injury initiation and in order to prevent or mitigate injury patterns seen in the spinal segments of this study, protective equipment or strategies should aim to reduce the coronal plane load and eccentricity to below the reported levels at injury initiation.

Segments of all sub-axial cervical spinal levels are grouped together here which likely affected measured failure loads since higher vertebrae are typically smaller and vertebral body size relates to compressive force at failure^28^. Injuries are also reported to all three vertebral structures despite calculating forces and moments only at the inferior vertebral disc.

Furthermore, only linear relationships were considered due to the limited experimental data available which is common for biomechanical testing studies of human tissue. More experiments, particularly with female specimens and focussing on the medium initial force eccentricity region, are warranted as they would allow for sex differences and potential nonlinear relationships between failure loads to be explored.

Finally, all experiments in this study resulted in specimen injury. The method of injury grading limited resolution of injury severity designation following the Abbreviated Injury Scale. Specimens were typically categorised as AIS 2 when vertebral fractures occurred and AIS 3 when an intervertebral disc rupture was sustained whereas the range of severity for these injuries in the real world likely ranges from AIS 2 to AIS 6 depending on the extent of fractures and involvement of the spinal cord which could not be considered within the available data. These limitations meant that injury risk curves could not be estimated based on current data and the MAIS. Tests at non-injurious and increasing severities with adapted injury severity scoring would allow for survival analysis and calculation of injury probability curves.

The data generated in this study provides the foundation for refining an injury criterion and tolerance values in compression with coronal plane bending. This provides targets for developing an appropriate mechanical neck surrogate for evaluating protective devices and strategies in vehicle crashes, falls and sports. The eccentricity-based criterion defined here describes an experimentally derived relationship between eccentricity and resultant coronal plane force at injury. Failure of cervical spine segments is expected at a resultant coronal plane force of 2998 N when eccentricity is 0 mm, with a linearly decreasing compressive coronal plane force tolerance at increasing eccentricity to when coronal plane force is low (250 N) failure would be expected at an eccentricity of 51.2 mm. The extreme levels of eccentricity at failure also indicate the types of injuries likely to be sustained, with low eccentricities producing a greater proportion of injuries to hard tissue structures while high eccentricities produce a greater proportion of injuries to soft tissue structures. No linear relationship between compressive force and lateral bending moment at injury was observed in the data, questioning the applicability of a lateral Nij type criterion for the cervical spine.

## Supplementary information

Injury Initiation Example Videos and Annotation.

## Data Availability

The experimental datasets are available from the corresponding author on reasonable request.

## References

[1] Krueger H, Noonan VK, Trenaman LM, Joshi P, Rivers CS (2013). The economic burden of traumatic spinal cord injury in Canada. Chroni. Dis. Inj. Can..

[2] Singh A, Tetreault L, Kalsi-Ryan S, Nouri A, Fehlings MG (2014). Global prevalence and incidence of traumatic spinal cord injury. Clin. Epidemiol..

[3] Badhiwala JH, Wilson JR, Fehlings MG (2019). Global burden of traumatic brain and spinal cord injury. Lancet Neurol..

[4] Wyndaele M, Wyndaele JJ (2006). Incidence, prevalence and epidemiology of spinal cord injury: what learns a worldwide literature survey?. Spinal Cord..

[5] King, A. I. Introduction to and Applications of Injury Biomechanics in *Accidental Injury:* Biomechanics *and Prevention* (eds. Yoganandan, N., Nahum, A. M. & Melvin, J. W.) (Springer Science+Business Media, 2015).

[6] Fung, Y. C. The Application of Biomechanics to the Understanding of Injury and Healing in *Accidental Injury:* Biomechanics *and Prevention* (eds. Nahum A. M. & Melvin J. W.) (Springer Science+Business Media, 2002).

[7] Alem, N., Nusholtz, G. & Melvin, J. Head and neck response to axial impacts. *Proceedings of the 28th Stapp Car Crash Conference*. 275–288 (1984).

[8] Carter JW, Ku GS, Nuckley DJ, Ching RP (2002). Tolerance of the cervical spine to eccentric axial compression. Stapp Car Crash J..

[9] Maiman DJ (1983). Compression injuries of the cervical spine: A biomechanical analysis. Neurosurgery..

[10] McElhaney, J. H., Paver, J. G., McCrackin, H. J. & Maxwell, G. M. Cervical spine compression responses. *Proceedings of the 27th Stapp Car Crash Conference*. 163–178 (1983).

[11] Nightingale, R. W. *et al*. The dynamic responses of the cervical spine: buckling, end conditions, and tolerance in compressive impacts. *Proceedings of the 41st Stapp Car Crash Conference*. 451–472 (1997).

[12] Nightingale RW, McElhaney JH, Richardson WJ, Myers BS (1996). Dynamic response of the head and cervical spine to axial impact loading. J. Biomech..

[13] Pintar, F. A. *et al*. Dynamic characteristics of the human cervical spine. *Proceedings of the 39th Stapp Car Crash Conference*. 195–202 (1995).

[14] Toomey DE, Yang KH, Yoganandan N, Pintar FA, Van Ee CA (2013). Toward a more robust lower neck compressive injury tolerance - An approach combining multiple test methodologies. Traffic Inj. Prev..

[15] Yoganandan N (1990). Injury biomechanics of the human cervical column. Spine..

[16] Yoganandan N, Sances A, Pintar F (1989). Biomechanical evaluation of the axial compressive responses of the human cadaveric and manikin necks. J. Biomech.Eng..

[17] Myers BS, Winkelstein BA (1995). Epidemiology, classification, mechanism, and tolerance of human cervical spine injuries. Crit. Rev. Biomed. Eng..

[18] Foster, J. B. *et al*. Analysis of cervical spine injuries and mechanisms for CIREN rollover crashes. *Proceedings of the 2012 International IRCOBI Conference on the Biomechanics of Injury*. 61–75 (2012).

[19] Ridella, S. & Eigen, A. Biomechanical Investigation of Injury Mechanisms in Rollover Crashes from the CIREN Database. *Proceedings of the 2008 International IRCOBI Conference on the Biomechanics of Impact*. 15 (2008).

[20] Digges, K. H. Summary report of rollover crashes. *2002, FHWA/NHTSA National Crash Analysis Centre* (Citeseer, 2002).

[21] National Highway Traffic Safety Administration. *Traffic Safety Facts 2017. A Compilation of Motor Vehicle Crash Data*. (National Highway Traffic Safety Administration, National Center for Statistics and Analysis, U.S. Department of Transportation, 2019).

[22] McMurry TL (2016). Epidemiology of moderate-to-severe injury patterns observed in rollover crashes. Accid. Anal. Prev..

[23] Parizel, P. M., Gielen, J. L. & Vanhoenacker, F. M. The Spine in Sports Injuries: Cervical Spine in *Imaging of Orthopedic* Sports Injuries. (eds. Vanhoenacker, F. M., Maas, M. & Gielen, J. L.) 377–389 (Springer Berlin Heidelberg, 2007).

[24] Hayes KC, Kakulas BA (1997). Neuropathology of Human Spinal Cord Injury Sustained in Sports-related Activities. J.Neurotrauma..

[25] Soltis, S. An Overview of Existing and Needed Neck Impact Injury Criteria for Sideward Facing Aircraft Seats. *The Third Triennial International Aircraft Fire and Cabin Safety Research Conference*. 12 (2001).

[26] Toomey, D. E. *et al*. Exploring the role of lateral bending postures and asymmetric loading on cervical spine compression responses. *Proceedings of the ASME 2009 International Mechanical Engineering Congress & Exposition*. 375–382 (2009).

[27] Van Toen C, Street J, Oxland TR, Cripton PA (2015). Cervical spine injuries and flexibilities following axial impact with lateral eccentricity. Eur. Spine J..

[28] Van Toen C, Melnyk AD, Street J, Oxland TR, Cripton PA (2014). The effect of lateral eccentricity on failure loads, kinematics, and canal occlusions of the cervical spine in axial loading. J. Biomech..

[29] Van Toen, C. Y. Biomechanics of cervical spine and spinal cord injury under combined axial compression and lateral bending loading. PhD Thesis, University of British Columbia (2013).

[30] Melnyk, A. D. *et al*. The effect of compression applied through constrained lateral eccentricity on the failure mechanics and flexibility of the human cervical spine. *(submitted to J. Biomech. Eng.)* (2020). 10.1115/1.4047342 32451551

[31] Carter, J. W. Compressive cervical spine injury: The effect of injury mechanism on structural injury pattern and neurologic injury potential. PhD Thesis, University of Washington (2002).

[32] Edwards, W. T. Principles of cervical spine biomechanical testing in *Frontiers in Head and Neck Trauma: Clinical and Biomechanical* (ed Yoganandan N.) (IOS Press, 1998).

[33] Crowell RR (1993). Cervical injuries under flexion and compression loading. J. Spinal Disord..

[34] Shea M, Edwards WT, White AA, Hayes WC (1991). Variations of stiffness and strength along the human cervical spine. J. Biomech..

[35] Moroney SP, Schultz AB, Miller JAA (1988). Analysis and measurement of neck loads. J. Orthopaed. Res..

[36] Panjabi MM (2001). Mechanical properties of the human cervical spine as shown by three-dimensional load-displacement curves. Spine.

[37] Moroney SP, Schultz AB, Miller JAA, Andersson GBJ (1988). Load-Displacement properties of lower cervical spine motion segments. J. Biomech..

[38] Yoganandan N, Chirvi S, Voo L, Pintar FA, Banerjee A (2018). Role of age and injury mechanism on cervical spine injury tolerance from head contact loading. Traffic Inj. Prev..

[39] Yoganandan N, Bass CR, Voo L, Pintar FA (2017). Male and Female Cervical Spine Biomechanics and Anatomy: Implication for Scaling Injury Criteria. J. Biomech. Eng..

[40] Maiman DJ, Yoganandan N, Pintar FA (2002). Preinjury cervical alignment affecting spinal trauma. J. Neurosurg. Spine.

[41] Toomey, D. E. Cervical spine tolerance and response in compressive loading modes including combined compression and lateral bending. Ph.D. Thesis, Wayne State University (2013).

[42] Van Toen C, Carter JW, Oxland TR, Cripton PA (2014). Moment Measurements in Dynamic and Quasi-Static Spine Segment Testing Using Eccentric Compression are Susceptible to Artifacts Based on Loading Configuration. J. Biomech. Eng..

[43] Orlowski KF, Bundorf RT, Moffatt EA (1985). Rollover Crash Tests-The Influence of Roof Strength on Injury Mechanics. SAE Transations..

[44] Lessley DJ (2014). Occupant Kinematics in Laboratory Rollover Tests: PMHS Response. Stapp Car Crash J..

[45] Newell RS, Blouin J-S, Street J, Cripton PA, Siegmund GP (2013). Neck posture and muscle activity are different when upside down: a human volunteer study. J. Biomech..

[46] Newell RS, Blouin J-S, Street J, Cripton PA, Siegmund GP (2018). The neutral posture of the cervical spine is not unique in human subjects. J. Biomech..

