# Supplementary material for: A neck compression injury criterion incorporating lateral eccentricity
Source: Sci Rep. 2020 Apr 28;10:7114. doi: 10.1038/s41598-020-63974-w (PMC7189232; doi:10.1038/s41598-020-63974-w)
Supplement: Supplementary file 1 — Injury Initiation Example Videos and Annotation. [file 41598_2020_63974_MOESM1_ESM.zip › Injury Initiation Examples - Annotation of high-speed video.pdf]

A neck compression injury criterion incorporating lateral eccentricity

Tom Whyte, Angela D. Melnyk, Carolyn Van Toen, Shun Yamamoto, John Street, Thomas R. Oxland, Peter A. Cripton.

Supplementary material – Identification of injury initiation

The supplementary video clips show high-speed footage of the dynamic tests, imaged at 18 kHz. These clips indicate examples of injury initiation as annotated in the still images below.

For specimen H1005 C6-T1, sudden abrupt motion can be seen on the anterior aspect of the specimen (see Figure S1), typical of hard tissue injury.

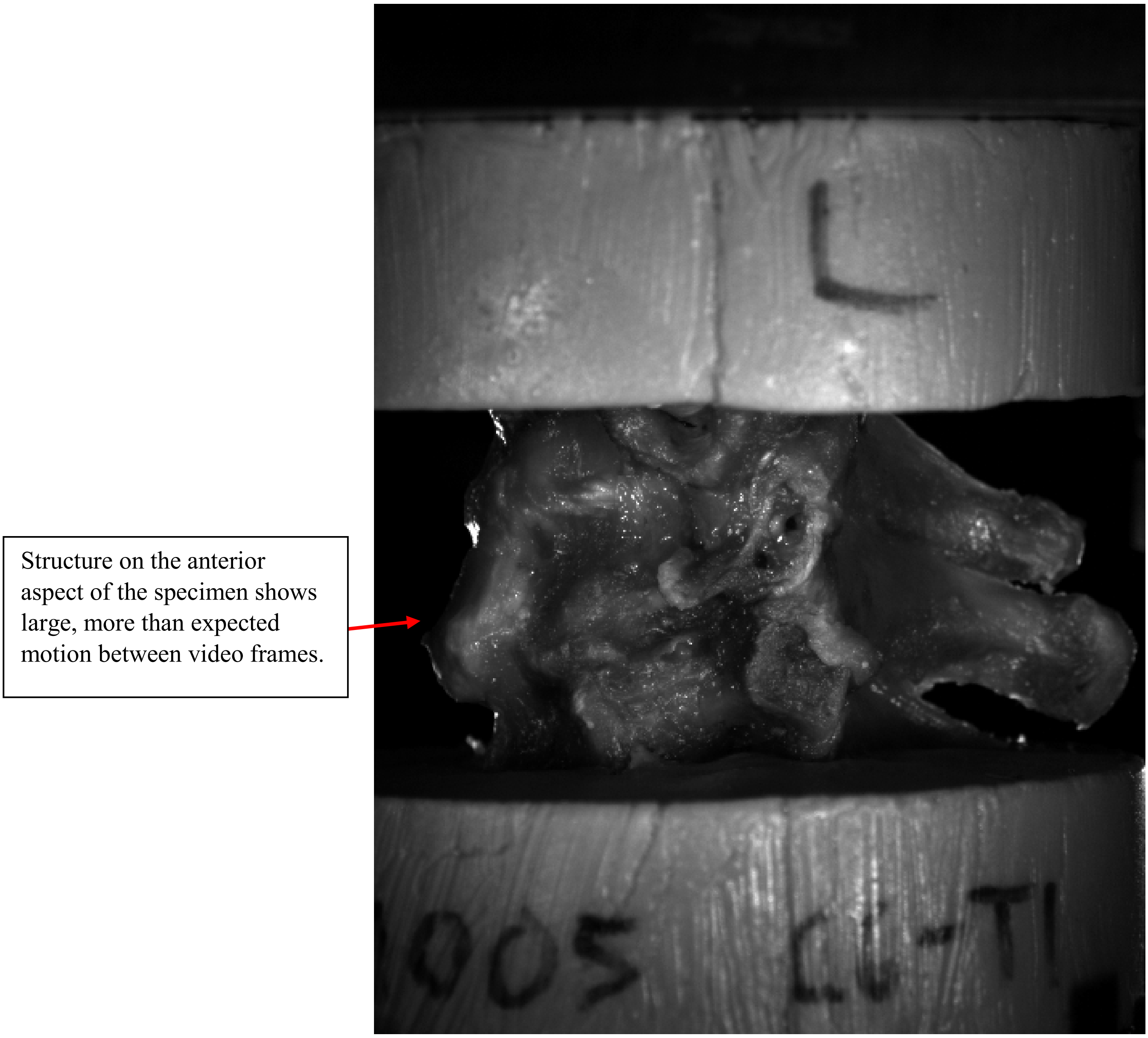

Figure S1 Still image from high-speed video of dynamic test of specimen H1005 C6-T1 indicating the structure on the anterior of the specimen that exhibits sudden large motion typical of hard tissue failure.

For specimen H1010 C5-C7, a sudden unexpected change in appearance can be seen in the superior facet capsule (see Figure S2), indicating initiation of soft tissue injury.

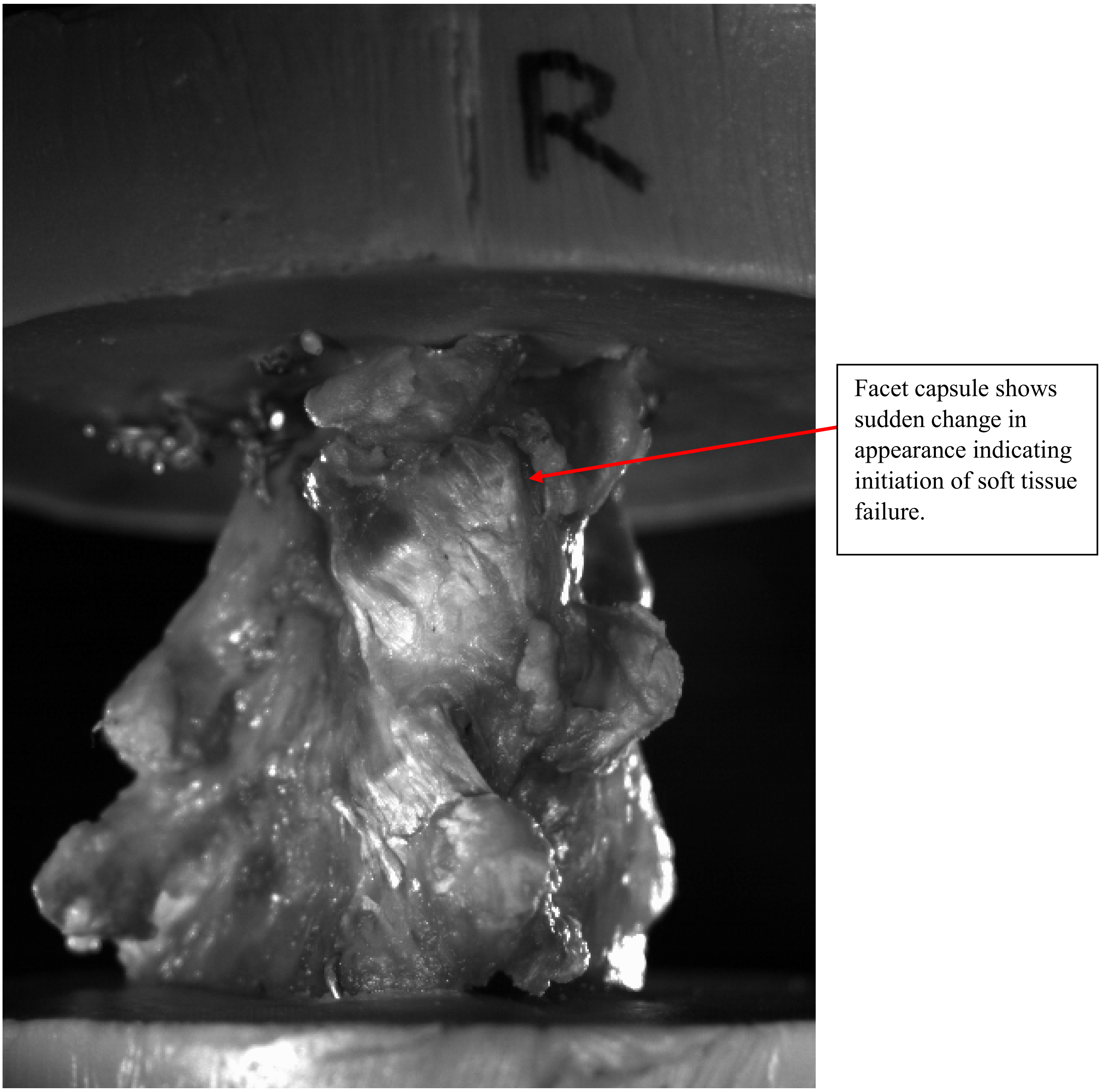

Figure S2 Still image from high-speed video of dynamic test of specimen H1010 C5-C7 indicating the superior facet capsule that exhibits a sudden change in appearance at initiation of soft tissue failure.
